# MicroRNA Dysregulation in Liver and Pancreas of CMP-Neu5Ac Hydroxylase Null Mice Disrupts Insulin/PI3K-AKT Signaling

**DOI:** 10.1155/2014/236385

**Published:** 2014-08-28

**Authors:** Deug-Nam Kwon, Byung-Soo Chang, Jin-Hoi Kim

**Affiliations:** ^1^Department of Animal Biotechnology, Konkuk University, Hwayang-dong, Kwangjin-gu, Seoul 143-701, Republic of Korea; ^2^Department of Cosmetology, Hanseo University, Seosan, Chungnam 356-706, Republic of Korea

## Abstract

CMP-Neu5Ac hydroxylase (*Cmah*)-null mice fed with a high-fat diet develop fasting hyperglycemia, glucose intolerance, and pancreatic *β*-cell dysfunction and ultimately develop characteristics of type 2 diabetes. The precise metabolic role of the* Cmah* gene remains poorly understood. This study was designed to investigate the molecular mechanisms through which microRNAs (miRNAs) regulate type 2 diabetes. Expression profiles of miRNAs in* Cmah*-null mouse livers were compared to those of control mouse livers. Liver miFinder miRNA PCR arrays (*n* = 6) showed that eight miRNA genes were differentially expressed between the two groups. Compared with controls, seven miRNAs were upregulated and one miRNA was downregulated in* Cmah*-null mice. Specifically, miR-155-5p, miR-425-5p, miR-15a-5p, miR-503-5p, miR-16-5p, miR-29a-3p, and miR-29b-3p were significantly upregulated in the liver and pancreas of* Cmah*-null mice. These target miRNAs are closely associated with dysregulation of insulin/PI3K-AKT signaling, suggesting that the* Cmah*-null mice could be a useful model for studying diabetes.

## 1. Introduction

Sialic acids are terminal components of glycoconjugate carbohydrate chains and are involved in cell-cell recognition, ligand-receptor, and cell-pathogen interactions [1–4]. The most common mammalian sialic acids are* N*-acetylneuraminic acid (Neu5Ac) and* N*-glycolylneuraminic acid (Neu5Gc). Neu5Gc is generated by hydroxylation of CMP-Neu5Ac to CMP-Neu5Gc, catalyzed by CMP-Neu5Ac hydroxylase (*Cmah*). An inactivating mutation in the* Cmah* gene during evolution led to humans losing the common mammalian cell surface molecule* N*-glycolylneuraminic acid (Neu5Gc) [3, 5, 6]. The mutated* Cmah* gene initiated a series of genetic and biochemical changes in the biology of sialic acids that may contribute to several unique aspects of human biology in health and disease [7, 8].

The effects of* Cmah* deactivation have been observed in* Cmah*-null mice. These mice have accumulation of Neu5Ac, which is also observed in humans. Similarly* Cmah*-null mice display human-like phenotypes including diminished acoustic sensitivity [9], altered startle response threshold (relative to normal mice) [9], hearing loss in old age [9], skin healing delay in adulthood [9], and a human-like muscular dystrophy phenotype following combined mutation of the* Dmd* gene [10]. Immunologically, these mice have enhanced B cell proliferation and antibody production, consistent with the observation that Neu5Gc is physiologically downregulated during normal B cell activation [11]. A recent study showed that high-fat diet (HFD)-fed* Cmah*-null mice exhibit fasting hyperglycemia, glucose intolerance, and pancreatic *β*-cell dysfunction that ultimately lead to the development of a type 2 diabetes-like syndrome [12]. Therefore, the HFD-fed* Cmah*-null mouse is considered one of the most suitable animal models of the human metabolic syndrome.

MicroRNAs (miRNAs) are small noncoding RNAs with lengths of approximately 22 nucleotides. miRNAs have essential roles in the regulation of diverse biological pathways and play an important role in insulin resistance in various tissues [13, 14]. For example, in obesity and diabetes, it has been demonstrated that miRNAs such as miR-375, miR-29, miR-320, miR-103, mir-107, and miR-126 play a crucial role in the regulation of glucose and lipid metabolism. This occurs through control of pancreatic islet cell function, adipocyte insulin resistance, hepatocyte insulin signaling, and glucose homeostasis [15–19]. miR-33a/b inhibits expression of insulin receptor substrate-2 (IRS-2) in hepatic cells, leading to reduced activation of downstream insulin signaling pathways including AKT and ERK. Antagonism of endogenous miR-33a/b upregulates fatty acid oxidation and the response to insulin in hepatocytes, suggesting this is a potential therapeutic target in the metabolic syndrome [20]. However, the precise molecular mechanisms by which miRNAs are involved in insulin resistance in* Cmah*-null mice remain obscure. The first aim of the present study was to identify changes in miRNA expression in the liver of* Cmah*-null mice. The second aim was to analyze miRNA expression profiles and to investigate the involvement of the insulin/PI3K-AKT signaling pathway.

## 2. Materials and Methods

### 2.1. *In Vivo* Experiments

All lines were maintained on a congenic C57Bl/6J background. The mice were allowed to eat and drink ad libitum and were fed standard mouse chow (Cargill Agri Purina, Inc., Seongnam-Si, Korea). Twelve-week-old wild type and* Cmah*-null male mouse in this study were used. All animal experiments were approved and performed under the guidelines of the Konkuk University Animal Care and Experimentation Community (IACUC approval number: KU12045).* Cmah* <tm1Ykoz> knockout mice were originally made and kindly provided by RIKEN.

### 2.2. MicroRNA Isolation and cDNA Generation

miRNA was isolated from mouse tissue using the miRNeasy mini kit (Qiagen, Valencia, CA, USA) according to the manufacturer's instruction. Concentration and purity of isolated miRNA were measured using the NanoDrop2000 spectrometer and samples were screened for an A260 : A280 ratio greater than 1.8. Conversion of miRNA to cDNA was carried out using 100 ng of miRNA according to the manufacturer's instructions for the RT^2^ miRNA First Strand Kit (SA Biosciences, Frederick, MD).

### 2.3. MicroRNA PCR Array Analysis

Changes in miRNA expression between samples were measured using a Liver miFinder miRNA PCR Array (SA Biosciences, Frederick, MD). Plates contained 84 of the most abundantly expressed and best-characterized miRNAs. The cDNA from individual samples was used as a template for the PCR array using SYBR green, according to the array instructions in an ABI ViiA 7 system (Applied Biosystems, Foster City, CA, USA). Data were analyzed using SABiosciences RT^2^ Profiler PCR Data Analysis software (available at http://pcrdataanalysis.sabiosciences.com/mirna/arrayanalysis.php) and were considered significant at >2.0 fold change. Relative quantitation of each miRNA was determined by normalizing to the small nuclear RNA housekeeping panel (*SNORD61*,* SNORD68*,* SNORD72*,* SNORD95*,* SNORD96A*, and* RNU6*-*2*) comparing the control wild type (WT) and* Cmah*-null samples using the 2-ΔΔCt method.

### 2.4. Real-Time Reverse Transcription Quantitative Polymerase Chain Reaction (RT-qPCR)

The total RNA obtained from tissues (liver and pancreas) was reversely transcribed using the QuantiTect Reverse Transcription Kit (Qiagen, Valencia, USA), according to the manufacturer's instructions. To assess gene expression, real-time reverse transcription polymerase chain reaction was conducted using an ABI ViiA 7 system (Applied Biosystems) with SYBR Green as the double-stranded DNA-specific fluorescent dye (Bio-Rad, Hercules, CA, USA). The mouse* Gapdh* gene was used as an internal control to normalize the RT-qPCR efficiency and to quantify the expression of mRNAs in WT and* Cmah*-null tissues. After normalization with* Gapdh*, the relative expressions of mRNA in* Cmah*-null tissues were compared with controls. RT-qPCR was performed for each sample in triplicate. The following primers were used: 5′-TCCAAGGGCTACACCAAATC-3′ and 5′-GTTTTCGAGGGCAGAGACTG-3′ for* Mapk3;* 5′-CTACACCCCATGCCTTCACT-3′ and 5′-GCTGAAGGTGAGGCTGATTC-3′ for* Raf1;* 5′-AGTGCGTGCAGAAGGAGATT-3′ and 5′-CACAACTTCTCGGCAGTCAA-3′ for* Ccnd2;* 5′-CCAGCCTGGCTATTTAGCTG-3′ and 5′-CCCAACTCAACTCCACCACT-3′ for* IRS1;* 5′-ACAACCTATCGTGGCACCTC-3′ and 5′-GACGGTGGTGGTAGAGGAAA-3′ for* IRS2;* 5′-CCCTTCTACAACCAGGACCA-3′ and 5′-ATACACATCCTGCCACACGA-3′ for* AKT1;* 5′-GTGAACACCATGCCTCACAC-3′ and 5′-CACAGTCCAAGCGCTCAATA-3′ for* FOXO1*. Eight miRNA primer sets (Mm_miR-155_1 miScript Primer Assay (MS00001701) for miR-155-5p, Mm_miR-425_2 miScript Primer Assay (MS00012012) for miR-425-5p, Mm_miR-15a_1 miScript Primer Assay (MS00001281) for miR-15a-5p, Mm_miR-503_1 (MS00002597) for miR-503-5p, Mm_miR-16_2 (MS00037366) for miR-16-5p, Mm_miR-29a_1 (MS00001372) for miR-29a-3p, Mm_miR-29b_1 (MS00005936) for miR-29b-3p, and Mm_miR-880_2 (MS00033103) for miR-880-3p) were obtained from Qiagen (Valencia, CA, USA). To confirm miRNA expression, RT-qPCR was performed on an ABI ViiA 7 system according to standard protocol. Reactions were run in triplicate.

### 2.5. Western Blot Analysis

Approximately 40 mg of tissue was homogenized using a Tissue-lyser (Qiagen) in ice cold RIPA buffer (with protease inhibitor cocktail) and centrifuged at 13,000 g for 15 min at 4°C and the pellet discarded. Protein concentration was measured using BCA Protein Assay Kit (Thermo Scientific, Seoul, Korea) and BSA was used to generate a standard curve. Proteins were electrophoresed on 10% SDS-PAGE gels under nonreducing conditions and were then transferred to PVDF membranes. Blots were incubated with primary antibodies for IRS2 (1 : 1000, Cell signaling, MA, USA), PI3K (1 : 1000, Cell signaling, MA, USA), AKT (1 : 1000, Cell signaling, MA, USA), and p-AKT (1 : 1000, Cell signaling, MA, USA), followed by incubation with a secondary goat anti-rabbit IgG antibody conjugated to horseradish peroxidase (HRP) (1 : 5000, Calbiochem, CA, USA). Bands were visualized using an ECL detection system (Amersham Pharmacia Biotech). An antiactin antibody was used to verify equal protein loading. Band intensity was quantified using ImageJ v1.32.

### 2.6. Bioinformatics Analysis for Putative MicroRNA Targets and Pathway

miRNA target prediction and pathway analysis was performed using DIANA-mirPath. This allowed identification of molecular pathways potentially altered by the expression of single or multiple miRNAs (http://diana.imis.athena-innovation.gr/DianaTools/index.php?r=mirpath/index) [21]. The software performs enrichment analysis of multiple miRNA target genes comparing each set of miRNA to all available pathways provided by the Kyoto Encyclopedia of Genes and Genomes (KEGG). Priority scores (enrichment *P* value) are assigned based upon the predicted strength of the miRNA interactions with components of the target pathway.

### 2.7. Immunohistochemistry (IHC)

For IHC, pancreatic tissues fixed in neutral buffer with 10% formalin were used. The tissues were embedded in paraffin and were sectioned at 3–5 *μ*m. Sections used for the quantification of histopathology were stained with Hematoxylin QS to provide backgrounds. All photomicrographs were acquired using a Zeiss Axiophot microscope (Carl Zeiss, Oberkochen, Germany) equipped with an Olympus DP70 high-resolution digital microscope camera (Olympus, Center Valley, PA). The Sniper and Hematoxylin QS were all purchased from Vector Laboratories (Burlingame, CA).

### 2.8. Transfection with miRNA Mimic

Hsa-miR-15a-5p mimic, hsa-miR-29a-3p mimic, hsa-miR-29b-3p mimic, hsa-miR-1 mimic, and AllStar negative control siRNA were purchased from Qiagen (Valencia, CA, USA). HepG2 (liver hepatocellular carcinoma) cell line was incubated in Dulbecco's modified Eagle's medium (DMEM, Invitrogen, Carlsbad, CA, USA) with 10% FBS. Cells were washed with PBS and the transfection was performed by using HiPerFect Transfection Reagents according to manufacturer's protocol (Qiagen, Valencia, CA). The final concentrations of the transfectants (hsa-miR-15a-5p mimic, hsa-miR-29a-3p mimic, and hsa-miR-29b-3p mimic) and their respective controls (AllStar siRNA for negative control and hsa-miR-1 mimic for positive control) were either 10 nM (miRNA mimic) or 50 nM (siRNAs). After 4 h, transfection medium was replaced by fresh cell culture medium and cells were incubated for another 48 h. For further analyses cells were then harvested by trypsin/EDTA.

## 3. Results

### 3.1. Altered miRNA Expression in the Liver and Pancreas of* Cmah*-Null Mice

**Figure 1:**
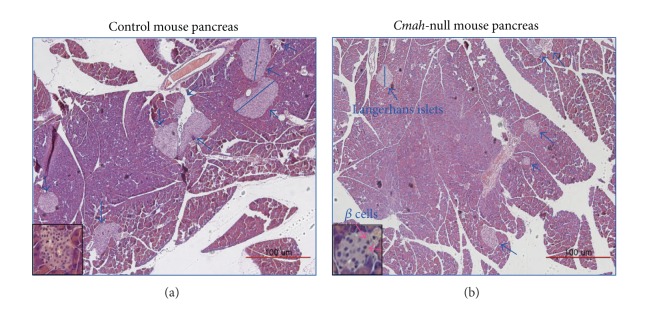
Representative pancreas tissues from control and* Cmah*-null mice. Islet size and number are significantly reduced in* Cmah*-null mouse compared to that of control mouse. Insert panels demonstrate that *β*-cell numbers in pancreas islets of* Cmah*-null mouse (b) were significantly lower than those of control (a).

To minimize differences in age, genetic background, sex, and time of sacrifice, which could affect metabolism, all animals with same age, sex, and genetic background were sacrificed at Zeitgeber time 9 (3 hrs before lights off). When* Cmah* homozygous mice with same age and sex from* Cmah* ^+/−^ ×* Cmah* ^+/−^ crosses were examined and weighted, homozygous* Cmah*-null mice were indistinguishable from their wild type or heterozygous littermates in all respects. However, in both size and numbers of pancreatic islets in* Cmah*-null mice were significantly decreased (Figure 1). Also numbers of *β*-cells in pancreatic islets were significantly lower than those of control (insert panels of Figure 1). Consistent with a previous report [9, 11], however, germline inactivation of the* Cmah* gene has only a minor effect on development such as hearing loss in old age, skin healing delay in adulthood, and abnormal B-cell numbers. Taken together, these observations suggest that* Cmah*-null mice are closely associated with dysfunction of pancreatic islets or B-cells.

**Figure 2:**
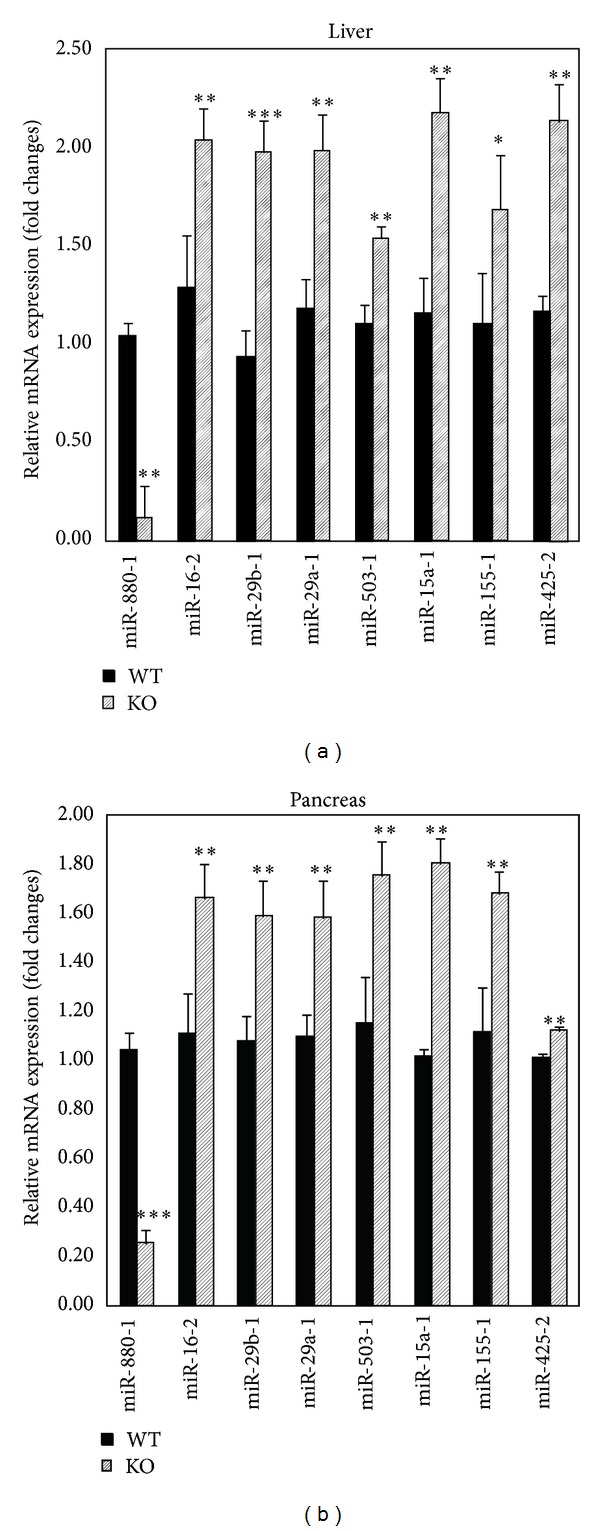
Validation of PCR array data by RT-qPCR. RT-qPCR analysis was performed using differentially expressed miRNAs in liver (a) and pancreas (b) RNA samples previously profiled by PCR array. Triplicate assays were carried out for each RNA sample and the relative amount of each miRNA was normalized to U6 snRNA. Data are expressed as fold changes of miRNA in livers and pancreas of* Cmah*-null versus control (C57BL/6) mice (mean ± SE, *n* = 3) (*P < 0.05; **P < 0.01; ***P < 0.001).

To examine differential expression of miRNAs between* Cmah*-null and control mouse livers, Liver miFinder miRNA PCR array analysis was performed (*n* = 6). Following array processing and normalization of raw array data, eight miRNAs were detected as differentially expressed. This included seven (87.5%; miR-16-5p, miR-29b-3p, miR-29a-3p, miR-503-5p, miR-15a-5p, miR-155-5p, and miR-425-5p) that were significantly upregulated and one (12.5%; miR-880-3p) that was downregulated (>2 folds, *P* < 0.05). To validate these findings, differentially expressed miRNAs identified by array were analyzed by RT-qPCR. The RT-qPCR data was consistent with the results of the array. This suggests that the data set obtained from array analysis accurately reflects the differential miRNA expression between the* Cmah*-null and control mice (Figure 2).

### 3.2. Identification of Putative Target Pathways Using* In Silico* Analysis

To identify the biological pathways overrepresented among the predicted targets of differentially expressed miRNAs in* Cmah*-null derived livers and pancreas, we examined pathway enrichment analysis by using DIANA-mirPath. The result showed putative pathways to be involved in a broad range of biological processes associated with the target genes (Table 1). Further, an investigation was carried out in statistically significant signaling pathways regulated by selected miRNAs. Among them, we found two major signal pathways such as insulin signaling (miR-155-5p, miR-425-5p, and miR-15a-5p) and PI3K-AKT signaling (miR-503-5p, miR-16-5p, miR-29a-3p, and miR-29b-3p) pathways (Table 2). The percentage of genes involved in the insulin/PI3K-AKT signaling pathway was higher than for other pathways. The biological processes in which these genes (*Pik3r1*,* Prkar2a*,* Sos1*,* Pik3r3*,* Fasn*,* Exoc7*,* Mapk3*,* Flot2*,* Raf1*, and* mTOR*) are involved are shown in Table 2.

### 3.3. *Cmah*-Null Mice Have Defects in the Insulin/PI3K-AKT Signaling Pathway

**Figure 3:**
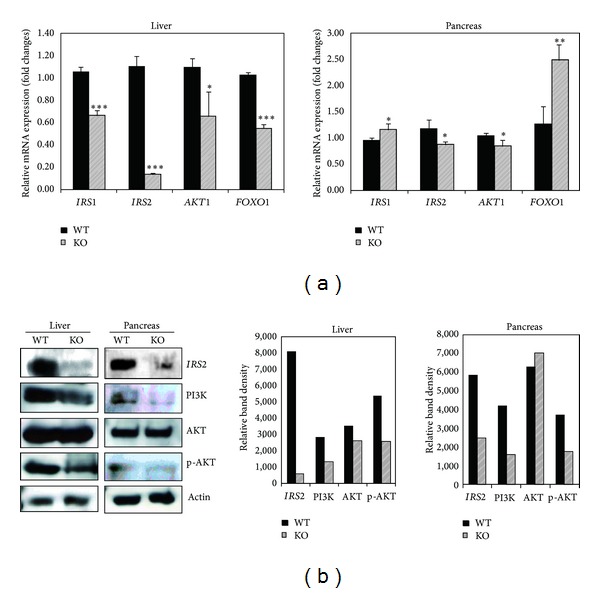
Expression changes in the insulin/PI3K-AKT signaling pathway. (a) Expression levels of* IRS1*,* IRS2*,* AKT1*, and* FOXO1* mRNAs in livers (left) and pancreas (right) of control and* Cmah*-null mice were determined using real-time reverse transcription PCR (RT-qPCR) (*P < 0.05; **P < 0.01; ***P < 0.001). (b) Western blot analysis of proteins involved in the target pathway in liver (left) and pancreas (right) of control and* Cmah*-null mice. Actin was used as a loading control. Band intensities were quantified by image processing and analysis was made using ImageJ v1.32.

To determine whether the loss of* Cmah* was responsible for differential expression of miRNAs, RT-qPCR and western blot analysis were performed using the liver and pancreas of* Cmah*-null and control mice.* Cmah*-null livers had significant downregulation of* IRS2*,* AKT*, and* Foxo1* mRNA expression, whilst* Foxo1* mRNA expression was significantly upregulated in the pancreas (Figure 3(a)). PI3Ks are composed of a catalytic subunit (p110) and a regulatory subunit. Various isoforms of the catalytic subunit (p110α, p110*β*, p110*γ*, and p110*δ*) have been isolated, and the regulatory subunits that associate with p110α, p110*β*, and p110*δ* are p85α and p85*β*. Among them, we used the p110α isoform of PI3K, because it plays a fundamental role in insulin signaling and control of hepatic glucose and lipid metabolism [22]. In addition, Akt is activated by phospholipid binding and activation loop phosphorylation at Thr308 by PDK1 and by phosphorylation within the carboxy terminus at Ser473. Therefore, we used the p-AKT (Phospho-Akt [Thr308]) antibody in this study.

**Figure 4:**
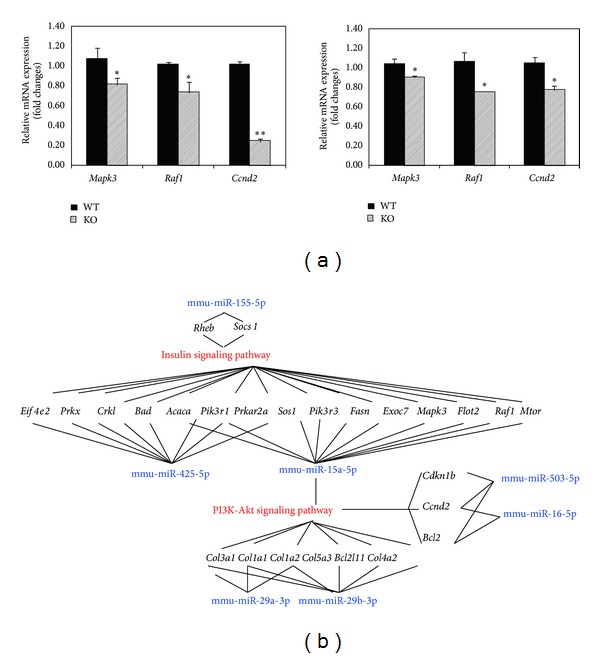
Identification of target genes of dysregulated miRNAs in* Cmah*-null mice. (a) RT-qPCR analysis was performed using randomly selected genes (*Mapk3*,* Raf1*, and* Ccnd2*), which involved the insulin/PI3K-AKT signaling pathway. Expression of mRNAs in livers (left) and pancreas (right) of control and* Cmah*-null mice were downregulated (*P < 0.05; **P < 0.01). (b) Dysregulated miRNAs and their target genes which involved in insulin/PI3K-AKT signaling pathway in liver and pancreas of* Cmah*-null mouse. Red and blue present the refined pathways and putative miRNAs, respectively. Black indicates putative target genes for their pathway.

RT-qPCR and western blotting analysis confirmed that mRNA and protein levels of IRS2, PI3K, and p-AKT were reduced in livers from* Cmah*-null mice (Figures 3(a) and 3(b)). This suggests that dysregulation of miRNA may affect both mRNA stability and protein translation. To confirm this possibility, we analyzed mRNA expression of genes involved in insulin/PI3K-AKT signaling by RT-qPCR. Expression of three randomly selected genes was significantly lower in* Cmah*-null mice than in controls (Figure 4(a)). As shown in Figure 4(b), we proposed an interaction of miRNAs with key components of the insulin/PI3K-AKT signaling pathway. This putative insulin/PI3K-AKT signaling pathway suggested that functional loss of Cmah gene during human evolution is closely associated with Type II diabetes due to altered miRNAs and target genes interaction.

### 3.4. Expression Validation of Target Genes by Using Three Different miRNAs

**Figure 5:**
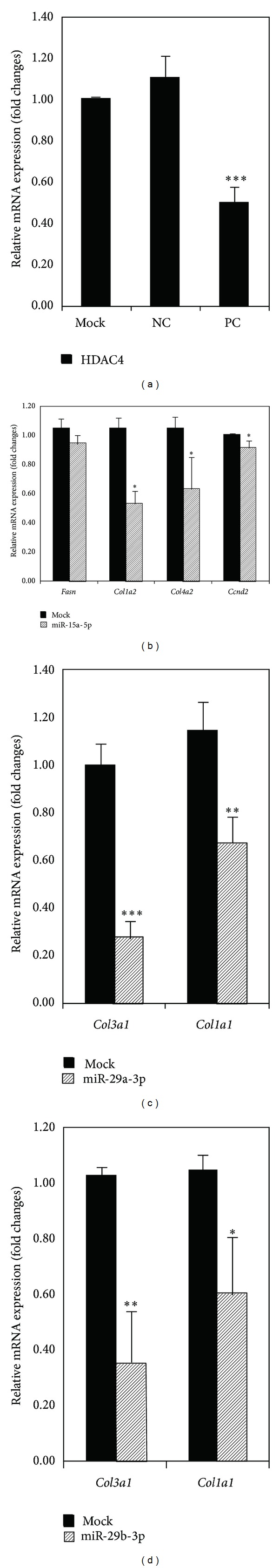
Posttranscriptional regulation of identified target genes by dysregulated miRNAs (miR-15a or miR-28a and b). (a) HDAC4 transcript level as a positive or negative control was analyzed by RT-qPCR after transfection using 10 nM miR-1 mimic or AllStar negative control siRNA. Mock: no transfection of miRNA mimic, NC: negative control (transfection of 50 nM AllStar negative control siRNA), PC: positive control (transfection of 10 nM miR-1 mimic). (b) RT-qPCR analysis of miR-15a predicted target genes. The transfection of miR-15a-5p mimic into HepG2 cells downregulates putative target gene mRNAs expression. (c) and (d) RT-qPCR analysis of miR-29a or -29b predicted target genes. The transfections of miR-29a-3p mimic or miR-29b-3p mimic into HepG2 cells downregulate their putative target gene mRNAs expression, respectively. The experiments were independently repeated three times and GAPDH served as internal control. (*P < 0.05; **P < 0.01; ***P < 0.001).

To determine whether the miRNAs dysregulate expression of their predicted target genes, we transfected 3 different types of miRNA mimics (hsa-miR-15a-5p mimic, hsa-miR-29a-3p mimic, and hsa-miR-29b-3p mimic) and 1 positive (hsa-miR-1 mimic) or negative control siRNA (AllStar negative control siRNA) into HepG2 cells. At 48 hrs after transfection, each cell was harvested and was provided for RT-qPCR analysis. In this study, the hsa-miR-1 mimic and AllStar siRNA were used as positive control and negative control for miRNA mimic experiments, respectively. A positive control, has-miR-1 mimic, markedly decreased the expression of HDAC4 in HepG2, compared to negative control group, whereas a negative control do not altered expression of HDAC4 gene (Figure 5(a)). We next determined the effect of miR-15a on mRNA expression of* Fasn*,* Col1a2*,* Col4a2*, and* Ccnd2* genes. Transfection of HepG2 cells with miR15a mimic significantly decreased the mRNA level of* Col1a2*,* Col4a2*, and* Ccnd2*, whereas* Fasn* mRNA expression was not changed (Figure 5(b)). Furthermore, HepG2 cells transfected with miR-29a and miR-29b mimic significantly downregulated its target mRNA expression such as Col3a1 and Col1a1 genes, compared to those of mock group (Figures 5(c) and 5(d)). Taken together, our observation strongly demonstrated that miRNAs dysregulation in* Cmah*-null mouse-derived liver could impair insulin/PI3K-AKT expression signaling during transition from glucose tolerance to intolerance.

## 4. Discussion

This study observed that expression of the miRNAs miR-155-5p, miR-425-5p, miR-15a-5p, miR-503-5p, miR-16-5p, miR-29a-3p, and miR-29b-3p in the liver of* Cmah*-null mice may downregulate components of the insulin/PI3K-AKT signaling pathway in concert with other genes. These include* Rheb*,* Socs1*,* Eif4e2*,* Prkx*,* Crkl*,* Bad*,* Acaca*,* Pik3r1*,* Prkar2a*,* Sos1*,* Pik3r3*,* Fasn*,* Exoc7*,* Mapk3*,* Flot2*,* Raf1*,* Mtor*,* Col3a1*,* Col1a1*,* Col1a2*,* Col5a3*,* Bcl2l11*,* Col4a2*,* Cdkn1b*,* Ccnd2*, and* Bcl2*.

miRNAs play a key role in regulating many physiological and pathological processes in humans, including diabetes and the related metabolic syndrome. It has been well demonstrated that miRNAs such as miR-375, miR-29, miR-320, miR-103, miR-107, and miR-126, play a crucial role in regulating glucose and lipid metabolism through control of pancreatic islet cell function, adipocyte insulin resistance, hepatocyte insulin signaling, and glucose homeostasis [15–19]. In this study, differential miRNA expression was found in* Cmah*-null mice liver using Liver miFinder miRNA PCR array. The majority of miRNAs differentially affected included genes encoding components of the insulin/PI3K-AKT signaling pathway. As the liver consumes more oxygen at rest than other organs and is thought to play a role in the systemic control of glucose and lipid utilization [23], gene expression and metabolic measurements in the liver were studied. These metabolic pathways are partly regulated at the transcriptional level, perturbation of which leads to pathological changes. In livers of* Cmah*-null mice, a rapid increase in miRNA expression was observed. Consistent with these results, insulin/PI3K-AKT signaling-related gene expression was significantly decreased. These results suggest that the key molecules altered in the pathway in* Cmah*-null mice are associated with a specific group of miRNAs.

Heterogeneous genetic background and lifestyle make it difficult to study the mechanism(s) of metabolic syndrome in humans. Presently, mouse models are a popular tool used to study human metabolic disorder as they provide evidence that can be extrapolated to validate the human condition. Because the phenotype of HFD-fed* Cmah*-null mice is similar to that of patients with type 2 diabetes, the* Cmah*-null mouse is one of the most suitable animal models of metabolic syndrome. It is not known, however, how impaired glucose tolerance and *β*-cell dysfunction arise in HFD-fed* Cmah*-null mice. To fill the gap of the phenotype of mice in relation to the previous observations [12], differential miRNA expression was examined in livers from control and* Cmah*-null mice using a pathway-focused glucose metabolism PCR array (data not shown). In Kyoto Encyclopedia of Genes and Genomes (KEGG), the molecular pathway analysis identified miRNAs in the upregulated or downregulated genes. Each case was linked to at least two different cellular pathways even if cross-linking was observed at low levels. Network-1 shows insulin-related signaling pathway (Table 2 and Figure 4(b) and includes 17 genes:* Rheb*,* Socs1*,* Pik3r1*,* Eif4e2*,* Prkar2a*,* Prkx*,* Crkl*,* Sos1*,* Bad*,* Acaca*,* Pik3r3*,* Fasn*,* Exoc7*,* Mapk3*,* Flot2*,* Raf1*, and* Mtor*. Network-2 functions in PI3K-AKT signaling pathway (Table 2 and Figure 4(b) and includes 19 genes:* Pik3r1*,* Prkar2a*,* Sos1*,* Pik3r3*,* Fasn*,* Exoc7*,* Mapk3*,* Flot2*,* Raf1*,* Mtor*,* Col3a1*,* Col1a1*,* Col1a2*,* Col5a3*,* Bcl2l11*,* COl4a2*,* Cdkn1b*,* Ccnd2*, and* Bcl2*. These data suggest that simultaneous impairment of insulin processes required for normal liver function could contribute to the development of metabolic disorders such as hyperglycemia and diabetes in* Cmah*-null mice.

Pirola et al. [24] suggested that liver cells mimic the effects of hyperinsulinemia, leading to downregulation of both PI3K/AKT signaling pathways and glucose uptake via a decrease in IRS-1/2 docking molecules. Because PI3K is the key molecule controlling the decrease in IRS-1/2 as downregulation of IRS-1/2 is effectively prevented by PI3K inhibition [24]. The same mechanism could be involved in liver cells of* Cmah*-null mouse. Definitely, for IRS-1/2, a PI3K-dependent pathway, with PI3K itself acting as an IRS-1 serine/threonine kinase, appears to be involved, although IRS-2 degradation is controlled by a PI3K-mTOR-dependent mechanism. Moreover, tissue-specific mechanisms may control IRS degradation, as IRS-1 degradation in adipocytes appears to be mTOR-dependent [24, 25]. The degradation of IRS proteins is promoted by serine/threonine phosphorylation and provides a molecular link to insulin resistance [26, 27].

In this study, mRNA and protein levels of IRS2 in the livers of* Cmah*-null mice were significantly downregulated. As a result,* IRS1* and* IRS2* downstream,* AKT1* and* mTOR* mRNA expression is also downregulated. As shown in Figure 4(b) miR-155-5p miR-15a-5p, and miR-425-5p in the case of insulin signaling and miR-29b-3p, miR-29a-3p, miR-16-5p, and miR-503-5p in the case of PI3K-AKT1-mTOR signaling were significantly dysregulated. This suggests that an insulin-dependent pathway in* Cmah*-null mice causes the increased glucose production. Previous studies showed that mice bearing a human-specific* Cmah* genetic mutation developed an obesity-related metabolism and diabetes [12]. It was shown that sialylation with Neu5Gc plays an important role in insulin production by *β* cells and that its loss contributes to *β*-cell dysfunction in mice. Taken together, these studies highlight the potential of the* Cmah*-null mouse to enhance scientific understanding of human metabolic syndrome.

## 5. Conclusion

Differential miRNA expression was found in* Cmah*-null mice liver using Liver miFinder miRNA PCR array. Among them, miR-155-5p, miR-425-5P, miR-15a-5p, miR-503-5p, miR-16-5p, miR-29a-3p, and miR-29b-3p were significantly upregulated in the liver and pancreas of* Cmah*-null mice. These target miRNAs are closely associated with dysregulation of insulin/PI3K-AKT signaling, suggesting that the* Cmah*-null mice could be a useful model for studying diabetes.

## Figures and Tables

**Table 1:** Target genes of dysregulated miRNAs identified in *Cmah*-null mice.

miRNA	KEGG pathway	*P* value	Target genes
miR-155-5p, **miR-15a-5p**, let-7c-5p, miR-106b-5p, let-7b-5p, miR-27b-9p	Acute myeloid leukemia	0.0280	8
**miR-15a-5p, miR-425-5p**	Aldosterone-regulated sodium reabsorption	0.0143	4
**miR-29b-3p, miR-29a-3p**, miR-24-3p	Amoebiasis	0.0000	6
**miR-155-5p, miR-503-5p, miR-16-5p, miR-425-5p**	Apoptosis	0.0008	6
miR-140-5p, let-7b-5p, miR-24-3p	Axon guidance	0.0007	9
**miR-15a-5p**, let-7b-5p	B cell receptor signaling pathway	0.0029	7
**miR-29a-3p**	Bacterial invasion of epithelial cells	0.0296	1
**miR-425-5p**	Carbohydrate digestion and absorption	0.0091	4
**miR-15a-5p, miR-425-5p**, miR-24-3p, miR-23b-3p, miR-27b-13p	Chagas disease (American trypanosomiasis)	0.0020	9
miR-335-5p,** miR-15a-5p**, let-7c-5p, let-7b-5p, **miR-425-5p**, miR-23b-3p, miR-27b-3p, miR-27a-3p	Chronic myeloid leukemia	0.0061	10
**miR-425-5p**	Circadian rhythm	0.0032	3
**miR-503-5**p,** miR-15a-5p**, let-7c-5p, **miR-16-5p**, let-7b-5p, miR-425-5p, miR-24-3p, miR-23b-3p, miR-27b-6p	Colorectal cancer	0.0007	9
**miR-29b-3p, miR-29a-3p**, miR-27a-3p	Cysteine and methionine metabolism	0.0012	2
**miR-503-5p**, let-7a-5p, **miR-29b-3p**	Cytokine-cytokine receptor interaction	0.0002	3
**miR-155-5p**	Cytosolic DNA-sensing pathway	0.0052	2
let-7d-5p, **miR-425-5p**, miR-24-3p	Dopaminergic synapse	0.0045	9
**miR-15a-5p**	Dorsoventral axis formation	0.0105	3
**miR-29b-3p, miR-29a-3p**	ECM-receptor interaction	0.0000	5
miR_142-3p,** miR-425-5p**	Endocrine and other factor-regulated calcium reabsorption	0.0216	1
**miR-15a-5p**, let-7c-5p, let-7b-5p, **miR-425-5p**	Endometrial cancer	0.0012	6
**miR-155-5p**, miR-335-5p, miR-106b-5p	Epstein-Barr virus infection	0.0240	3
**miR-15a-5p**	Fatty acid biosynthesis	0.0000	1
**miR-15a-5p**	Fc gamma R-mediated phagocytosis	0.0103	7
**miR-15a-5p**, **miR-16-5p, miR-29b-3p, miR-29a-3p**, let-7b-5p, **mmu-miR-425-5p**	Focal adhesion	0.0105	12
miR_142-3p, let-7b-5p, **miR-425-5p**	GABAergic synapse	0.0216	9
miR_142-3p, **miR-15a-5p, miR-425-5p**	Gap junction	0.0230	7
miR_142-3p, **miR-425-5p**, miR-24-3p	Gastric acid secretion	0.0216	5
**miR-15a-5p**	Glycosaminoglycan biosynthesis-heparan sulfate/heparin	0.0000	3
**miR-15a-5p**	Glycosphingolipid biosynthesis-globo series	0.0209	2
**miR-15a-5p**, let-7b-5p, miR-24-3p	Glycosphingolipid biosynthesis-lacto and neolacto series	0.0004	3
let-7b-5p	Glycosylphosphatidylinositol (GPI)-anchor biosynthesis	0.0017	3
miR_142-3p, **miR-15a-5p**, miR-106b-5p, miR-24-3p	GnRH signaling pathway	0.0216	6
**miR-16-5p**, miR-106b-5p, **miR-425-5p**	Hedgehog signaling pathway	0.0454	5
miR-503-5p, miR-335-5p, **miR-15a-5p**, let-7c-5p, **miR-16-5p**, miR-106b-5p, miR-20a-5p, let-7b-5p, **miR-425-5p**	Hepatitis B	0.0008	14
**miR-155-5p, miR-15a-5p**, miR-106b-5p, miR-20a-5p, let-7b-5p	Hepatitis C	0.0339	9
**miR-155-5p**	Herpes simplex infection	0.0145	3
**miR-503-5p**, miR-199a-5p, **miR-15a-5p**, miR-24-3p	HIF-1 signaling pathway	0.0175	2
**miR-155-5p**, miR-335-5p, let-7c-5p, miR-23b-3p, miR-27b-12p	HTLV-I infection	0.0145	3
**miR-155-5p, iR-15a-5p, miR-425-5p**	*Insulin signaling pathway *	0.0339	10
**miR-155-5p**, miR-140-5p	Intestinal immune network for IgA production	0.0339	2
**miR-15a-5p, miR-425-5p**, miR-24-3p	Long-term depression	0.0005	7
**miR-15a-5p**, miR-24-3p	Lysine degradation	0.0196	4
let-7d-5p, let-7b-5p, **miR-425-5p**	MAPK signaling pathway	0.0073	12
**miR-503-5p**, let-7a-5p	Measles	0.0435	2
miR-199a-5p, **miR-15a-5p, miR-425-5p**	mTOR signaling pathway	0.0006	6
**miR-155-5p**, miR-140-5p	NF-kappa B signaling pathway	0.0038	2
**miR-503-5p**	p53 signaling pathway	0.0047	2
**miR-155-5p**, **miR-503-5p**, miR-199a-5p, **miR-15a-5p**, let-7c-5p, **miR-16-5p**, miR-106b-5p, miR-20a-5p, let-7b-5p, **miR-425-5p**, miR-23b-3p, miR-27b-4p, miR-27a-3p	Pathways in cancer	0.0064	20
**miR-503-5p, miR-15a-5p, miR-16-5p, miR-29b-3p, miR-29a-3p**	*PI3K-AKT signaling pathway *	0.0060	18
**miR-29b-3p, miR-29a-3p**	Protein digestion and absorption	0.0000	5
miR_142-3p, miR-106b-5p, **miR-425-5p**, miR-24-3p	Retrograde endocannabinoid signaling	0.0207	7
**miR-15a-5p**, let-7b-5p	T cell receptor signaling pathway	0.0392	7
**miR-503-5p, miR-15a-5p**, let-7c-5p, miR-140-5p, miR-34c-5p, **miR-29b-3p**, miR-24-3p, miR-23b-3p, miR-27b-3p	TGF-beta signaling pathway	0.0000	6
**miR-155-5p**	Toll-like receptor signaling pathway	0.0000	4
**miR-155-5p, miR-503-5p, miR-16-5p**, miR-106b-5p, miR-20a-5p	Toxoplasmosis	0.0294	2
**miR-155-5p, miR-503-5p**, miR-196a-5p, let-7a-5p, let-7c-5p, miR-34c-5p, miR-27b-5p, miR-27a-3p	Transcriptional misregulation in cancer	0.0052	3
**miR-155-5p**	Tuberculosis	0.0072	3
miR_142-3p, **miR-15a-5p**	Vasopressin-regulated water reabsorption	0.0207	5
**miR-15a-5p, miR-425-5p**, miR-106b-5p	VEGF signaling pathway	0.0045	6
**miR-503-5p**, miR-335-5p	Viral carcinogenesis	0.0044	3
let-7c-5p, miR-140-5p, **miR-16-5p**, miR-24-3p, miR-23b-3p, miR-27b-11p	Wnt signaling pathway	0.0398	6

Bold text represents significantly downregulated miRNAs.

**Table 2:** KEGG pathway analysis of miRNA target genes.

miRNAs	Significant pathway	*P* value	Target genes
miR-155-5p, miR-15a-5p, miR-425-5p	Insulin signaling pathway	0.0339	*Rheb*, *Socs1*, *Pik3r1*, *Eif4e2*, *Prkar2a*, *Prkx*, *Crkl*, *Sos1*, *Bad*, *Acaca*, *Pik3r3*, *Fasn*, *Exoc7*, *Mapk3*, *Flot2*, *Raf1*, *Mtor *

miR-503-5p, miR-15a-5p, miR-16-5p, miR-29b-3p, miR-29a-3p	PI3K-Akt signaling pathway	0.0060	*Pik3r1*, *Prkar2a*, *Sos1*, *Pik3r3*, *Fasn*, *Exoc7*, *Mapk3*, *Flot2*, *Raf1*, *Mtor*, *Col3a1*, *Col1a1*, *Col5a3*, *Bcl2l11*, *COl4a2*, *Cdkn1b*, *Ccnd2*, *Bcl2 *

## Abbreviations

*Cmah*: CMP-Neu5Ac hydroxylase
HFD: High-fat diet
miRNAs: MicroRNAs
Neu5Ac: * N*-acetylneuraminic acid
Neu5Gc: * N*-glycolylneuraminic acid
KEGG: Kyoto Encyclopedia of Genes and Genomes
RT-qPCR: Real-time reverse transcription quantitative polymerase chain reaction.
